# Monocyte chemoattractant protein-1 from a conditioned medium of bone marrow-derived mesenchymal stem cells promotes bone regeneration by enhancing macrophage phenotype polarization

**DOI:** 10.1186/s40902-026-00503-1

**Published:** 2026-02-24

**Authors:** Kosuke Hashizume, Wataru Katagiri, Ryoko Takeuchi, Daisuke Suda, Tadaharu Kobayashi

**Affiliations:** 1 Division of Reconstructive Surgery for Oral and Maxillofacial Region, Faculty of Dentistry & Graduate School of Medical and Dental Science, Niigata University, Niigata, Japan; 2 Department of Oral and Maxillofacial Surgery, Gifu University Graduate School of Medicine, Gifu, Japan

**Keywords:** Bone regeneration, Conditioned medium, MCP-1, Macrophage phenotype polarization, Mesenchymal stem cell

## Abstract

**Background:**

We have reported that the cytokines and chemokines contained in conditioned medium of human mesenchymal stem cells (MSC-CM), which were derived from bone marrow, promote bone regeneration. We recently reported macrophage phenotype polarization towards the anti-inflammatory M2 phenotype induced by MSC-CM and its potential to establish regenerative condition and assist subsequent bone regeneration. However, the specific factors in the MSC-CM responsible for this process remain unclear. Monocyte chemoattractant protein (MCP) -1, present in MSC-CM, promotes cell migration and activation of the monocyte-macrophage lineage; therefore, we hypothesized that MCP-1 is one of the key factors in MSC-CM-induced macrophage phenotype polarization. The effect of MCP-1 on MSC-CM-induced macrophage phenotype polarization and subsequent bone regeneration was investigated in this study.

**Methods:**

MCP-1 was depleted from MSC-CM (depMSC-CM) and used in subsequent experiments. Rat bone marrow macrophages were incubated in MSC-CM or depMSC-CM and expression of macrophage markers was examined in vitro. In addition, the effect of MSC-CM and depMSC-CM on bone regeneration and macrophage phenotype polarization were evaluated using rat calvaria defect model in vivo.

**Results:**

MSC-CM enhanced M2 macrophage marker expression in rat bone marrow macrophages compared to those treated with depMSC-CM in vitro. In addition, MSC-CM increased the number of M2 macrophage marker-positive cells in bone defects and enhanced subsequent bone regeneration in a rat calvaria bone defect model.

**Conclusions:**

MCP-1 seemed to be a one of the most contributing factors in MSC-CM-induced macrophage phenotypic polarization and subsequent bone regeneration.

## Background

Autogenous or allogenic mesenchymal stem cell (MSC) implantation is one of the strategies in regenerative medicine in oral and maxillofacial regions. MSC implantation for bone regeneration is applied clinically based on the finding that MSCs from multiple tissues promote bone regeneration [1–3]. Although this approach is valuable in regenerative medicine, some issues such as tumorigenesis [4], poor survival rate of implanted cells [5, 6], and transmission of infectious diseases remain to be resolved. Furthermore, while bone regeneration therapy using stem cells in the oral and maxillofacial region is revolutionary, it is expensive, and alternative methods such as autogenous bone graft and artificial materials have developed, so it cannot be said to be widely used.

However, comparing bone regenerative therapies using stem cells, bone regenerative therapy using the conditioned medium of human mesenchymal stem cells which were derived from bone marrow (MSC-CM) is expected to complement the drawbacks of stem cell transplantation, as it is low-cost and does not involve cell transplantation.

Osugi et al. have shown not only that mixing MSC-CM with existing bone graft materials and transplanting it can promote early bone regeneration through enhanced osteoblast migration [7], but also that it can maintain osteoclast function in animal models of drug-induced osteonecrosis of the jaw [8]. MSC-CM also supports extracellular matrix reconstruction and promotes cartilage regeneration and mandibular condylar resorption [9].

MSC-CM contains numerous factors that promote bone regeneration. Insulin like growth factor-1 (IGF-1), vascular endothelial growth factor-A (VEGF), and transforming growth factor-β1 (TGF-β1) in MSC-CM promote cell migration, angiogenesis, and cell differentiation; they accelerate bone regeneration [7]. Furthermore, we considered that not only the effects of these cytokines, but also the anti-inflammatory effects of MSC-CM and the creation of a regenerative environment are important for early bone regeneration.

Katagiri et al. have reported that MSC-CM elicit macrophage phenotype polarization and contribute to the establishment of an anti-inflammatory milieu during the early phases of bone regeneration [10].

Macrophages are activated by diverse stimuli and categorized into two major subtypes according to their functions. Classically activated M1 macrophages produce pro-inflammatory cytokines, whereas alternatively activated M2 macrophages release anti-inflammatory cytokines and growth factors. Recent studies have revealed that MSC-CM affects macrophage activation and subsequently creates an anti-inflammatory milieu that promotes tissue regeneration [11–13]. Furthermore, a previous study showed that monocyte chemoattractant protein (MCP)-1, suggested to be one of the factors that promote macrophage phenotype polarization, is present in MSC-CM by cytokine antibody array analysis [14]. However, the direct contribution of M2 macrophages to the MSC-CM-induced early osteogenesis remains unclear.

MCP-1 is a cytokine that plays an important role in inflammatory responses, particularly activating the monocyte/macrophage lineage [15]. MCP-1 promotes inflammation by promoting cell migration and the production of inflammatory factors, supporting immune responses in the host.

In this study, the role of MCP-1 in MSC-CM-induced bone regeneration was investigated.

## Methods

### Preparation of conditioned medium

We used the commercially available human bone marrow-derived mesenchymal stem cells (hMSCs) (Lonza inc., Walkersville, MD, USA). hMSCs were cultured in a culture dish (100 × 20 mm) at 37℃ in 5% CO_2_ in Dulbecco’s modified Eagle’s medium (DMEM) (Gibco; Thermo Fisher Scientific, Waltham, Ma, USA) containing 10% fetal bovine serum (FBS) (Biowest, Nuaillé, France). After primary culture, the cells were subcultured at a density of 1 × 10^4^ cells/ cm^2^. hMSCs at the third to fifth passage and used in the experiments.

**Fig. 1:**
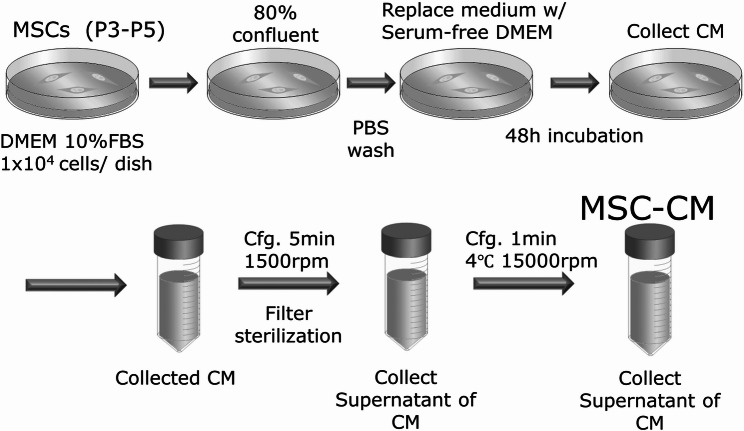
Schematic diagram of preparation process of MSC-CM

hMSCs that were about 80% confluent were re-fed with serum-free DMEM. After being incubated for 48 h, the cell-cultured conditioned medium was filtered through a 0.22 μm filter sterilizer. The collected, cultured conditioned medium was defined as MSC-CM (Fig. 1). MSC-CM was vacuum freeze-dried and stored at 4 or -80℃ before being used for the following experiments. The quality of MSC-CM is guaranteed by confirming the concentrations of cytokines such as IGF-1, TGF-β1 and VEGF that we have already reported as the important factors for bone regeneration [7].

### MCP-1 depletion from MSC-CM

MCP-1 was depleted from the MSC-CM using rabbit anti-human polyclonal antibodies against MCP-1 (ab9669; Abcam, Cambridge, UK). Briefly, Protein G magnetic beads (SureBeads Protein G; Bio-Rad, USA), pre-bound with 100 ng/mL anti-MCP-1 antibodies, were added to MSC-CM and mixed gently at 4℃ for 1 h. Antibody beads were magnetized, and the supernatant was collected. MCP-1 depletion was confirmed using MCP-1 enzyme-linked immunosorbent assay (ELISA) kit (DCP00; R&D, USA) and Quantikine immunoassay control group 1 (QC01-1; R&D) as a negative control, according to the manufacturer’s instructions. Depleted MSC-CMs were defined as depMSC-CM and used in subsequent experiments.

### Bone marrow macrophage isolation and activation

Bone marrow cells were isolated from the femurs of 8-week-old male Wistar rats (Japan SLC, Shizuoka, Japan) and plated on 60-mm cell culture dishes or cover slips. They were differentiated into bone marrow macrophages (BMMs) in DMEM supplemented with 20 ng/mL macrophage colony stimulating factor (Peprotech, NJ, USA) at 37℃ in 5% CO_2_ for 7 days and used in subsequent experiments.

### Immunocytochemical analysis

The cells were fixed in 4% Paraformaldehyde Phosphate Buffer Solution (PFA), Fetal bovine serum (Sigma-Aldrich), permeabilized in 0.1% Triton X-100 (Sigma-Aldrich), blocked in 5% goat serum, and incubated with primary antibodies against CD11b (1:1000; Ab1211, Abcam), inducible nitric oxide synthase (iNOS) (1:100; Ab15323, Abcam), and CD206 (1:10000; ab64693, Abcam). Next, secondary antibodies, AF647-conjugated anti rabbit (1:1000; Ab150079, Abcam), AF488-conjugated anti mouse (1:1000; Ab150113, Abcam) were used and cells were counterstained with DAPI (D9542, Sigma Aldrich). Samples were observed using a fluorescence microscope (Axioplan 2; Carl Zeiss AG, Oberkochen, Germany). The positive cell numbers within three randomly selected areas in each well were averaged to determine the positive cell rate for each well. Three different wells for each group were used to evaluate the data, and evaluation was done blindly by two researchers.

### Cell culture with MSC-CM or depMSC-CM

hMSCs were cultured with DMEM-10%FBS. After confluence, cell culture supernatant was refreshed and cultured with MSC-CM or depMSC-CM for 48 h.

BMMs were cultured with macrophage colony stimulating factors for 7 days as described above, then cell culture supernatant was refreshed and cultured with MSC-CM or depMSC-CM for 48 h.

### Real-time quantitative Reverse Transcriptase-Polymerase Chain Reaction (qRT-PCR)

hMSCs and BMMs were cultured with MSC-CMs, depMSC-CM, or DMEM(-) for 48 h, and total RNA was extracted using the RNeasy Mini kit (Qiagen N. V., Venlo, Netherlands). Reverse-transcribed into cDNA and qRT-PCR were performed using PrimeScript RT Master Mix and TB Green Premix Ex Taq II (TaKaRa Bio, Shiga, Japan) with a Thermal Cycler Dice Real-Time System III (TaKaRa Bio). Table 1 showes the sequences of primers of markers for M1 macrophages (*iNOS* and *CD80*), markers for M2 macrophages [*CD206* and Arginase-1 (*Arg-1*)], and osteogenesis-related genes [osteopontin (*OPN*), type I collagen (*COLⅠ*), alkaline phosphate (*ALP*), and osteocalcin (*OCN*)]. The obtained results were normalized to Glyceraldehyde-3-phosphate dehydrogenase (*GAPDH*) and the 2^-ΔΔCt^ method was used to calculate relative expression levels.

**Table 1:** Primer sequences used for qRT-PCR

Gene		Sequence	Accession no.
*OPN*	F	5'-ACACATATGATGGCCGAGGTGA-3'	NM_000582.2
	R	5'-GTGTGAGGTGATGTCCTCGTCTGTA-3'	
*COL I*	F	5'-CCCGGGTTTCAGAGACAACTTC-3'	NM_000088.3
	R	5'-TCCACATGCTTTATTCCAGCAATC-3'	
*ALP*	F	5'-GCCATTGGCACCTGCCTTAC-3'	NM_000478.5
	R	5'-AGCTCCAGGGCATATTTCAGTGTC-3'	
*OCN*	F	5'-CATGAGAGCCCTCACACTCCT-3'	NM_199173.5
	R	5'-CACCTTTGCTGGACTCTGCAC-3'	
*iNOS*	F	5'-GCTGCCAAGCTGAAATTGAATG-3'	NM_000625.4
	R	5'-TCTGTGCCGGCAGCTTTAAC-3'	
*CD80*	F	5'-CACCTCCATTTGCAATTGACC-3'	NM_005191.4
	R	5'-TCCTGCAAAGCAACTGAAGTGA-3'	
*CD206*	F	5'-ATGCCCGGAGTCAGATCACAC-3'	NM_002438.4
	R	5'-TTCTGCAGCACTTTCAATGGAAAC-3'	
*Arg-1*	F	5'-CTGGCAAGGTGGCAGAAGTC-3'	NM_000045.3
	R	5'-ATGGCCAGAGATGCTTCCAA-3'	
*GAPDH*	F	5'-AGGCTAGCTGGCCCGATTTC-3'	NM_001256799.2
	R	5'-TGGCAACAATATCCACTTTACCAGA-3'	

### Rat calvarial bone defect model

All animal experiments were performed in strict accordance with the protocols reviewed by the Animal Care and Use Committee of Niigata University (No. SA00456).

10-week-old male Wistar rats were anesthetized with an intraperitoneal injection of a mixture of medetomidine, midazolam, and butorphanol. After shaving the parietal region, a transverse incision was made posterior to the eyes, and a longitudinal incision was made at the left ear base. The periosteum was raised to expose the calvarial bones. Two calvarial bone defects, 5 mm in diameter, were created using a trephine bur (Dentech, Tokyo, Japan) and rinsed with PBS to remove bone debris. MSC-CM (30 µl), depMSC-CM (30 µL), or DMEM(-) (30 µL) were implanted to the bone defect using atelocollagen sponges (Terudermis1, Olympus Terumo Bio-materials Corp., Tokyo, Japan) as a scaffold. Finally, the periosteum and skin were sutured using a 4 − 0 nylon thread. The rats were sacrificed at 72 h, 1 week, and 2 weeks after implantation, and the specimens were harvested.

The rats were randomly divided into four groups. The experimental groups were as follows: MSC-CM group, depMSC-CM group, DMEM(-) group, or defect group (defect only) (*n* = 6 in each group at every time points).

### Microcomputed Tomography (micro-CT) analysis

Specimens from all groups were harvested at 72 h, 1 week, and 2 weeks after surgery and analyzed using a micro-CT system (CosmoScan Gx, Rigaku Co., Tokyo, Japan). The rats were anesthetized by an intraperitoneal injection of 4% chloral hydrate. The specimens were observed by micro-CT, and three-dimensional (3D) images were reconstructed using Analyze software (version 12.0; AnalyzeDirect Inc., KS, USA) with threshold values from 600 to 2000, which approximately corresponds to Hounsfield Units. Surgically created bone defects with a diameter of 5 mm were displayed from above, and bone regenerated inside a circle of 5 mm diameter was defined as newly formed bone. The newly formed bone area and its proportion relative to the bone defects were evaluated. The newly formed bone area was evaluated as a percentage of surgically created bone defects.

### Histological analysis

Specimens from all groups were harvested at 72 h, 1 week, or 2 weeks after implantation. The samples were fixed in 10% neutral formalin and decalcified with 10% EDTA (pH 7.4) for four weeks. Samples were dehydrated with graded ethanol, embedded in paraffin, and cut into 3 μm thickness in the coronal plane using a microtome (REM-710, YAMATO KOHKI Industrial Co., Ltd., Saitama, Japan). The sections were rehydrated, stained with hematoxylin and eosin (H&E), and analyzed under a light microscope (FX630, OLYMPUS Co., Tokyo, Japan).

### Immunohistochemical analysis

Immunohistochemical analysis for iNOS (1:100; Ab15323, Abcam) to evaluate M1 macrophages and for CD206 (1:10000; ab64693, Abcam) to detect M2 macrophages were performed. The sections were dewaxed, rehydrated, and antigen retrieval was performed with citrate buffer (pH 6.0) for 10 minutes at 121˚C. The sections were then incubated with 0.3% H_2_O_2_ in methanol for 30 min to block endogenous peroxidase activity. After washing with PBS, the sections were blocked for non-specific binding using 10% goat serum for 1 h at room temperature, and then incubated with the primary antibody overnight at 4˚C. Subsequently, the sections were reacted with EnVision Plus (Dako, CA, USA) for 1 h and developed with 3,3’-diaminobenzidine (DAB) solution. Finally, the sections were counterstained with hematoxylin after DAB staining. At 72 h, 1 week, and 2 weeks after transplantation, six specimens from each experimental group were investigated. Three areas with a diameter of 200 μm were randomly set at the edge of each bone defect, and the number of positive cells contained within the area was averaged to determine the number of positive cells for each specimen. Data was evaluated using *n* = 6 for each group and evaluation was done blindly by two researchers.

### Statistical analysis

All data are expressed as mean ± standard deviation (SD). The statistical analysis was performed using the Sigma-Plot software 15 (Grafiti LLC, Palo Alto, CA, USA). The differences among the experimental groups were examined by one-way analysis of variance (ANOVA) test using Tukey’s honesty significant test. A *P* value less than 0.05 was considered to be statistically significant.

## Results

### MCP-1 concentration in MSC-CM and depMSC-CM

The concentration of MCP-1 in MSC-CM, depMSC-CM, and DMEM(-) were 414.9 ± 138.2 pg/mL, 11.8 ± 1.3 pg/mL, and 5.2 ± 2.1 pg/mL, respectively. MSC-CMs contained significantly higher levels of MCP-1 than depMSC-CM or DMEM(-).

### MSC-CM regulated macrophage phenotype-related gene expression in BMMs

**Fig. 2:**
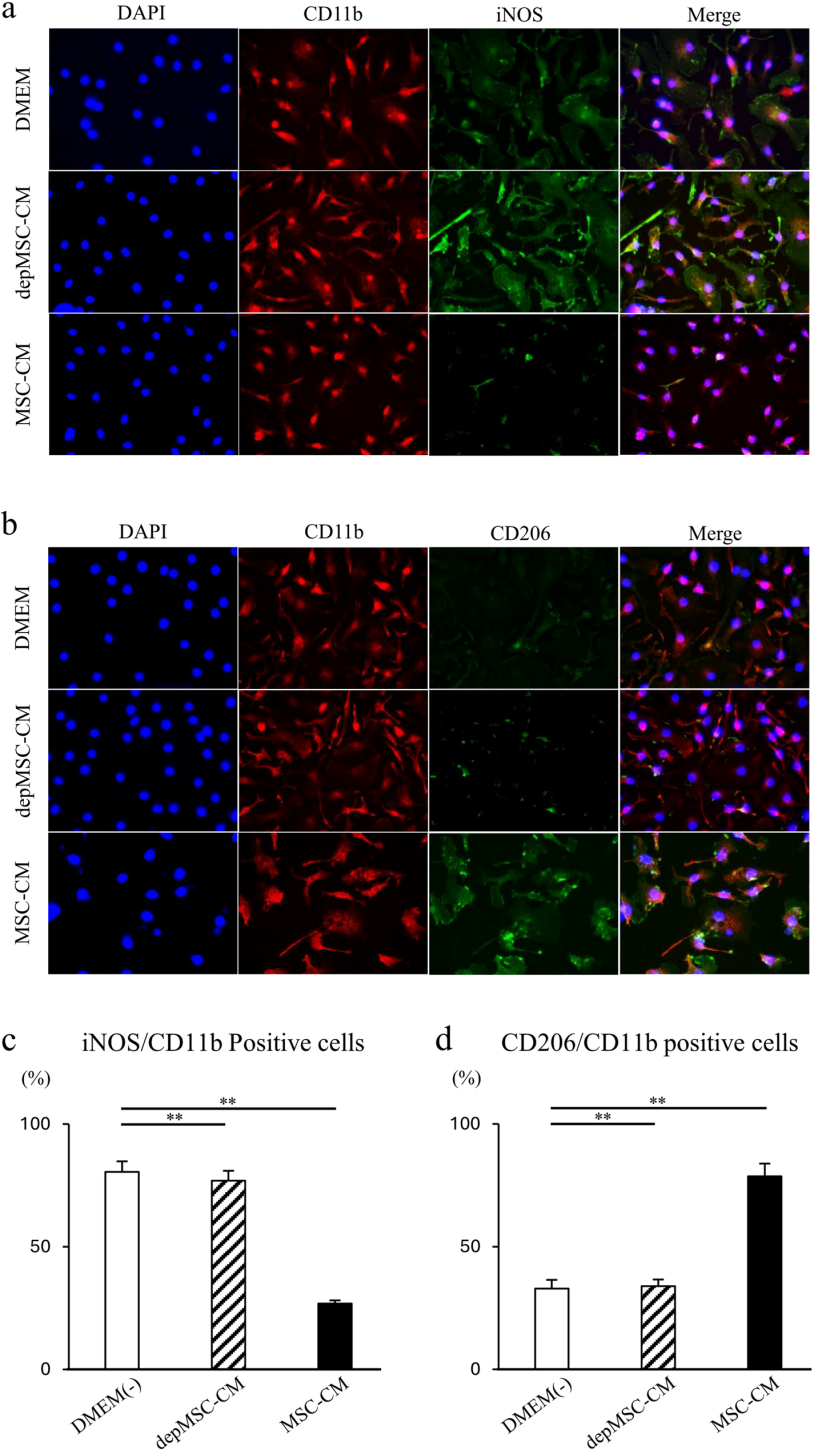
Effects of MSC-CM and depMSC-CM on BMM gene expression. Representative images of BMMs immunocytologically stained for CD11b (red), iNOS (green), DAPI (blue) (**a**), CD11b (red), CD206 (green), and DAPI (blue) (**b**) 48 h after incubation with MSC-CMs or depMSC-CMs. Scale bar: 50 μm. Ratios of iNOS and CD11b positive cells (**c**) and CD206 and CD11b positive cells (**d**)

Immunocytochemical analysis revealed that the percentage of CD11b and iNOS positive M1 cells decreased, while that of CD11b and CD206 positive M2 cells increased in MSC-CM-treated BMMs compared to that in depMSC-CM-treated BMMs (Fig. 2a-d).

**Fig. 3:**
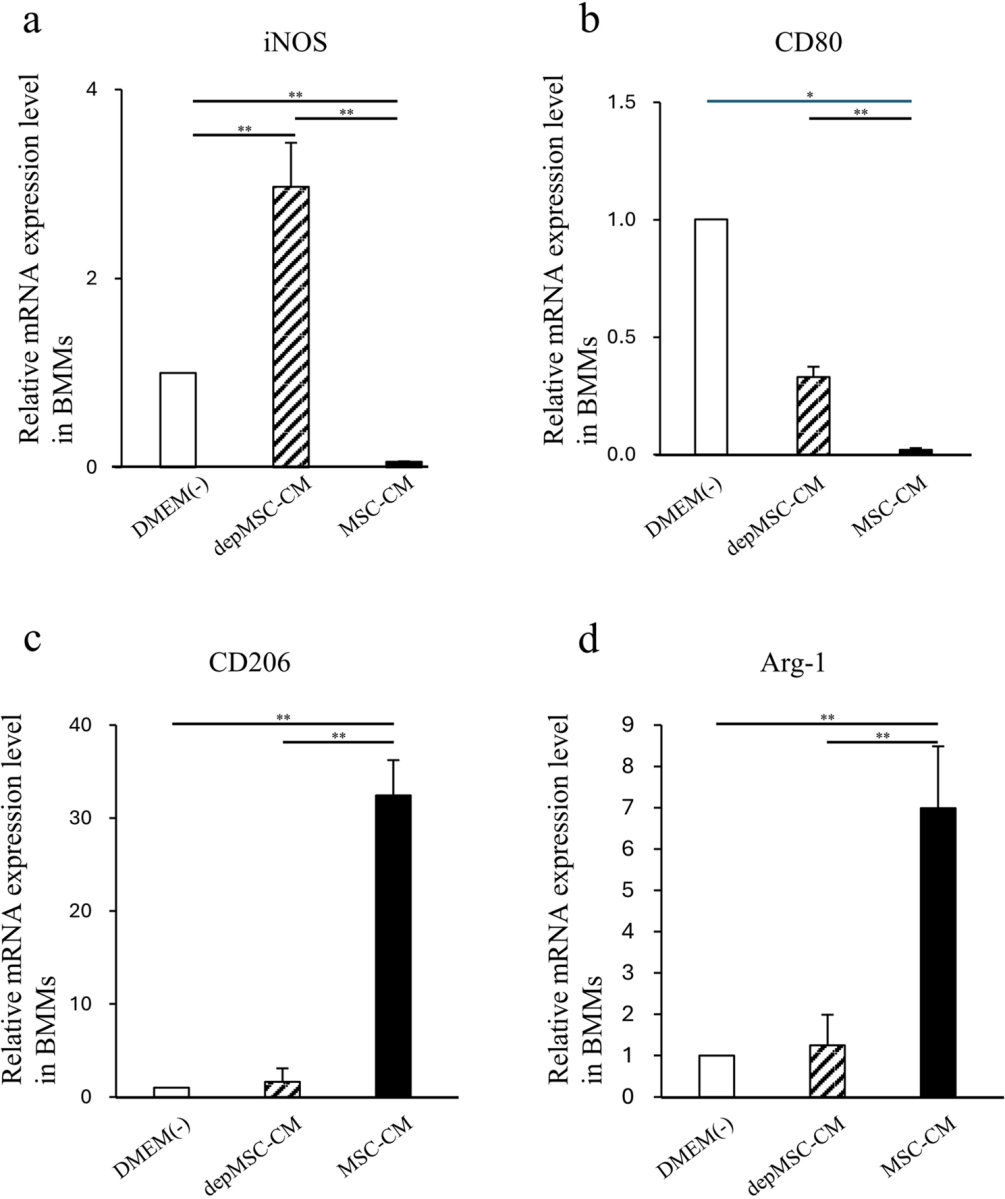
Effects of MSC-CM and depMSC-CM on BMMs gene expression. Gene expression in BMMs 48 h after incubation with MSC-CM and depMSC-CM (*iNOS* (**a**), *CD80* (**b**),* CD206* (**c**), *Arg-1* (**d**)). The results are expressed relative to the mRNA expression levels in DMEM(-)-treated cells (n = 3 per group). Data are represented as mean ± SD; *P < 0.05, **P < 0.01

Consistent with these results, qRT-PCR revealed that the expression levels of the pro-inflammatory M1 macrophage markers (*iNOS* and *CD80*) were significantly downregulated, and that the M2 macrophage markers (*CD206* and *Arg-1*) were significantly upregulated in BMMs cultured with MSC-CM compared to those cultured with depMSC-CM (*p* < 0.01) (Fig. 3a-d).

### MSC-CMs enhanced osteogenesis-related gene expression in hMSC

**Fig. 4:**
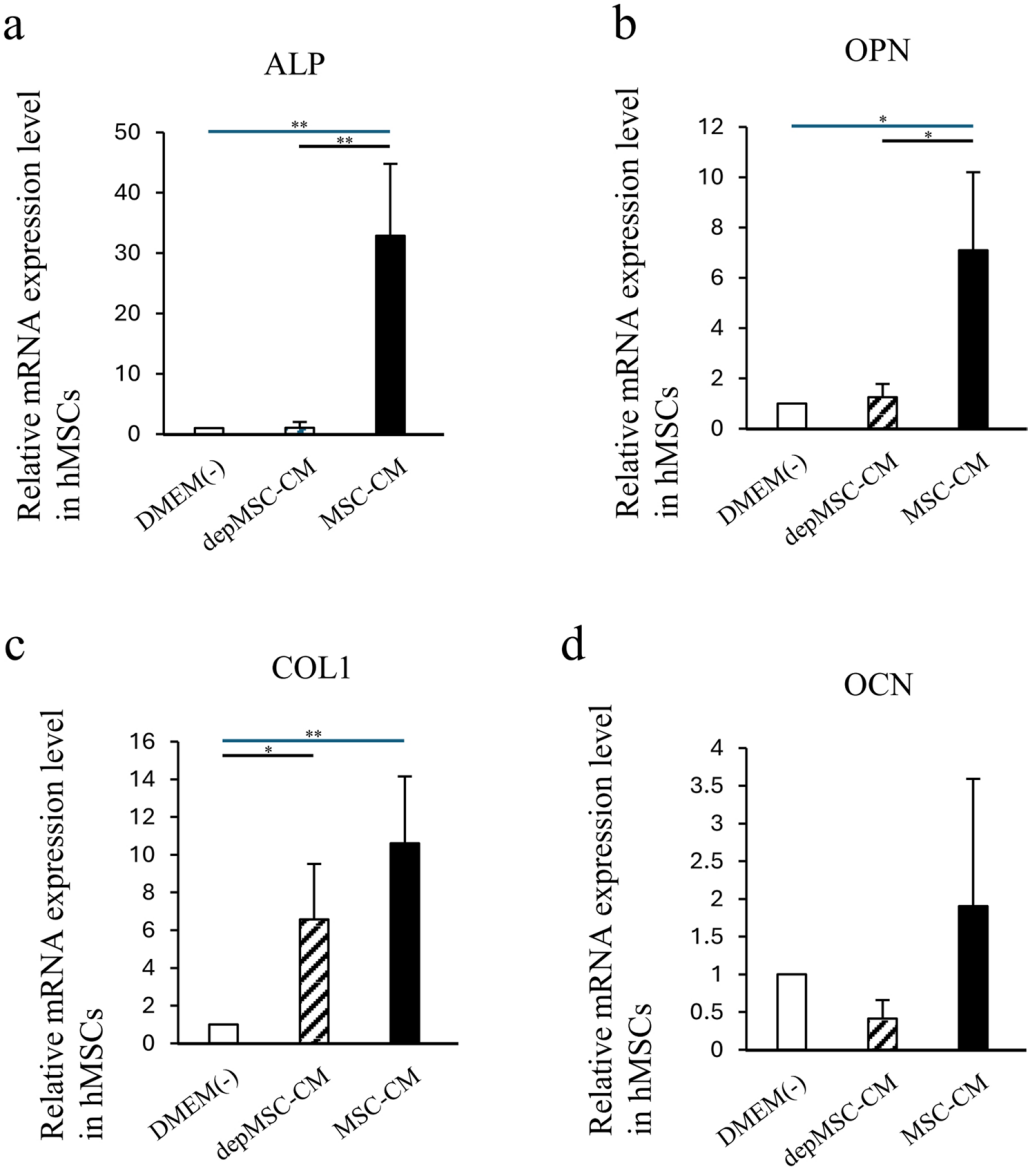
Effects ofEffects of MSC-CM and depMSC-CM on hMSC gene expression. Relative expression levels of osteogenesis-related genes (*ALP* (**a**),* OPN* (**b**), *COL I* (**c**), *OCN* (**d**)) in MSC- and depMSC-CM. The results are expressed relative to the mRNA expression levels in DMEM(-)-treated cells (n = 3 per group). Data are represented as mean ± SD; *P < 0.05, **P < 0.01

The expression levels of *ALP* and *OPN* were upregulated significantly in hMSC cultured with MSC-CM compared to those cultured with DMEM(-) and depMSC-CM (*p* < 0.01 and *p* < 0.05, respectively) (Fig. 4a, b). The expression of *COL I* was significantly higher in hMSCs cultured with MSC-CM compared to those cultured with DMEM(-) (*p* < 0.01) (Fig. 4c). However, there was no significant difference between those cultured with MSC-CM and depMSC-CM. MSC-CM enhanced *OCN* expression in hMSCs; however, the difference between the DMEM(-) and depMSC-CM groups was not statistically significant (Fig. 4d).

### MSC-CM enhanced bone regeneration compared to depMSC-CM

**Fig. 5:**
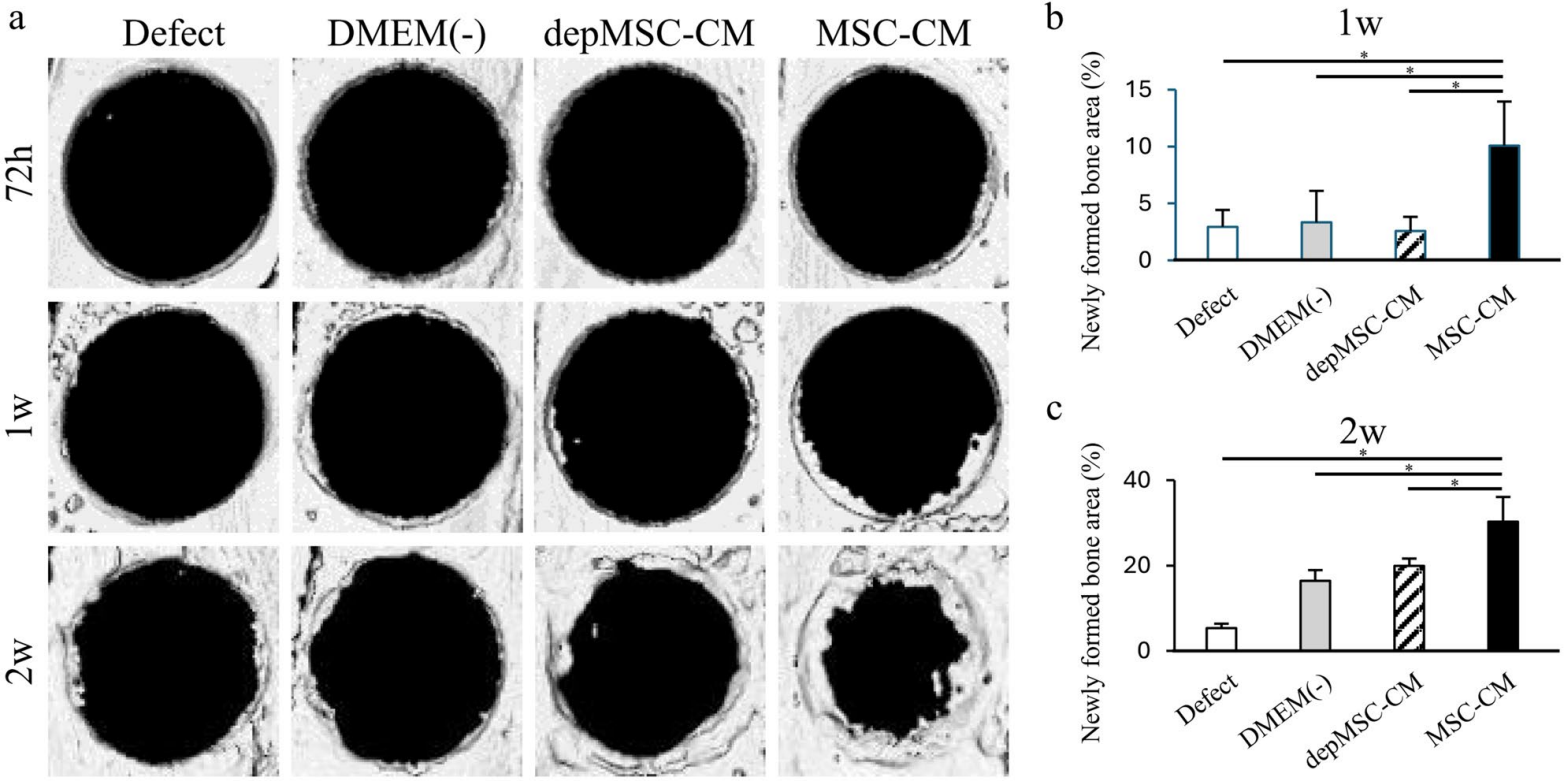
Micro-CT analysis of bone formation after implantation of MSC-CM and depMSC-CM. Reconstructed images of rat calvaria bone defects at 72 h, 1 week, and 2 weeks after implantation of MSC-CM, depMSC-CM, or DMEM(-) (**a**). The defect group did not undergo implantation. Areas with the newly regenerated bone were measured, indicating that MSC-CM significantly promoted bone regeneration at 1 and 2 weeks after implantation compared to the other groups (**b**, **c**). Data is represented as mean ± SD; *P < 0.01

Areas with the newly regenerated bone within the bone defect were evaluated using micro-CT scanning and three-dimensional images were reconstructed at 72 h, 1 week, and 2 weeks after implantation (Fig. 5a). Areas with the newly regenerated bone was calculated as a percentage of the graft area. Seventy-two hours after implantation, bone regeneration was not evident in any group. After 1 week, areas with the newly regenerated bone in the MSC-CM group (10.8 ± 3.9%) were significantly higher compared to those in the depMSC-CM (2.6 ± 1.2%), DMEM(-) (3.3 ± 2.8%), and defect (2.9 ± 1.5%) groups (Fig. 5b). After 2 weeks, areas with the newly regenerated bone in the MSC-CM group (30.3 ± 5.8%) were higher compared with the depMSC-CM (19.9 ± 1.7%), DMEM(-) (16.4 ± 2.5%), and defect (5.3 ± 1.0%) groups significantly (Fig. 5c).

**Fig. 6:**
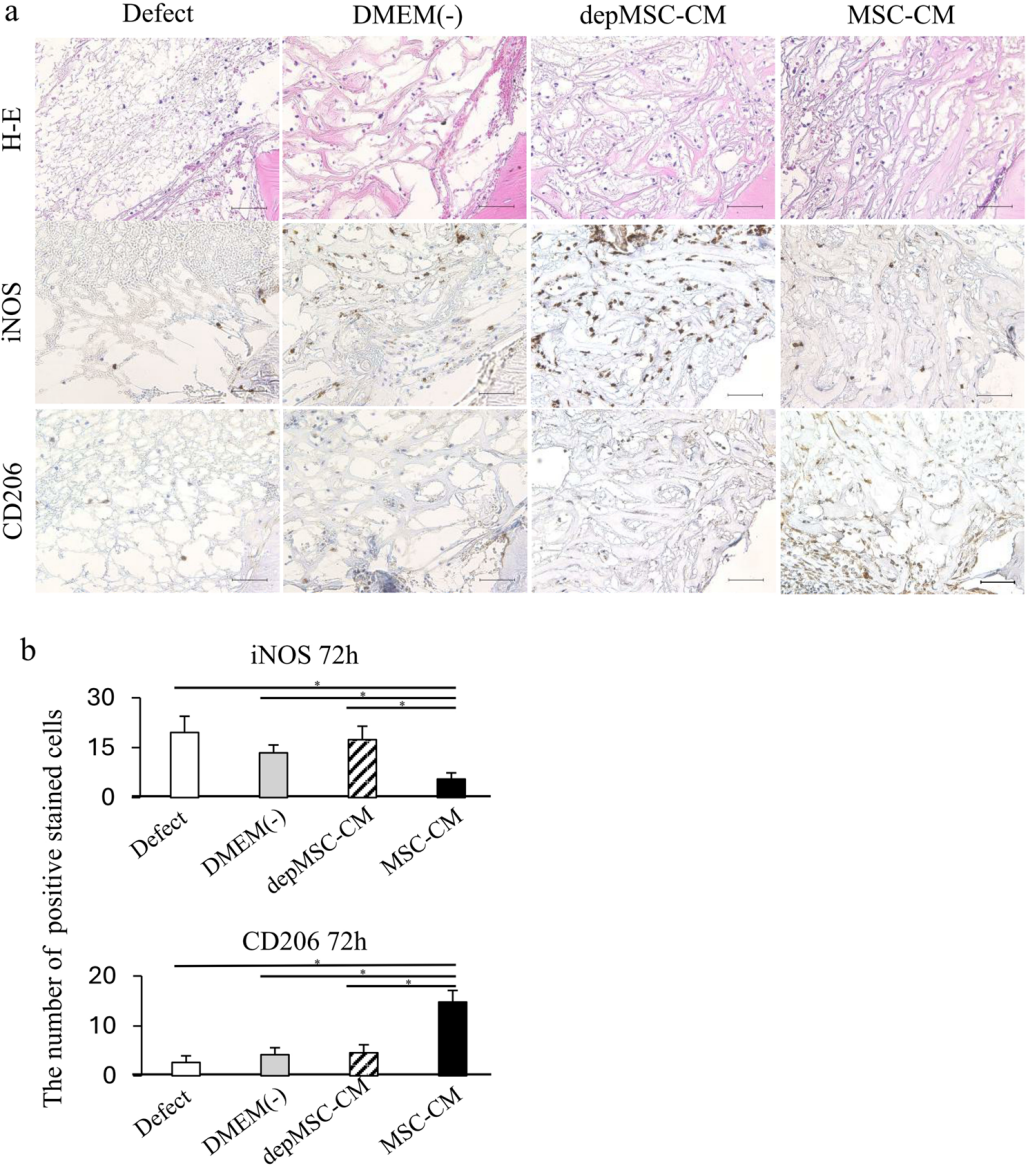
Immunohistochemistry for the newly regenerated bone 72 h after implantation. H&E staining revealed the orientation of the specimens, indicating newly formed bone edges and surrounding tissue (**a**). Seventy-two hours after implantation, iNOS-positive cells were few in the MSC-CM group than in the other groups, whereas CD206 positive cells was the highest among all groups (**b**). Scale bar: 50 μm. Data are represented as mean ± SD; *P < 0.01

**Fig. 7:**
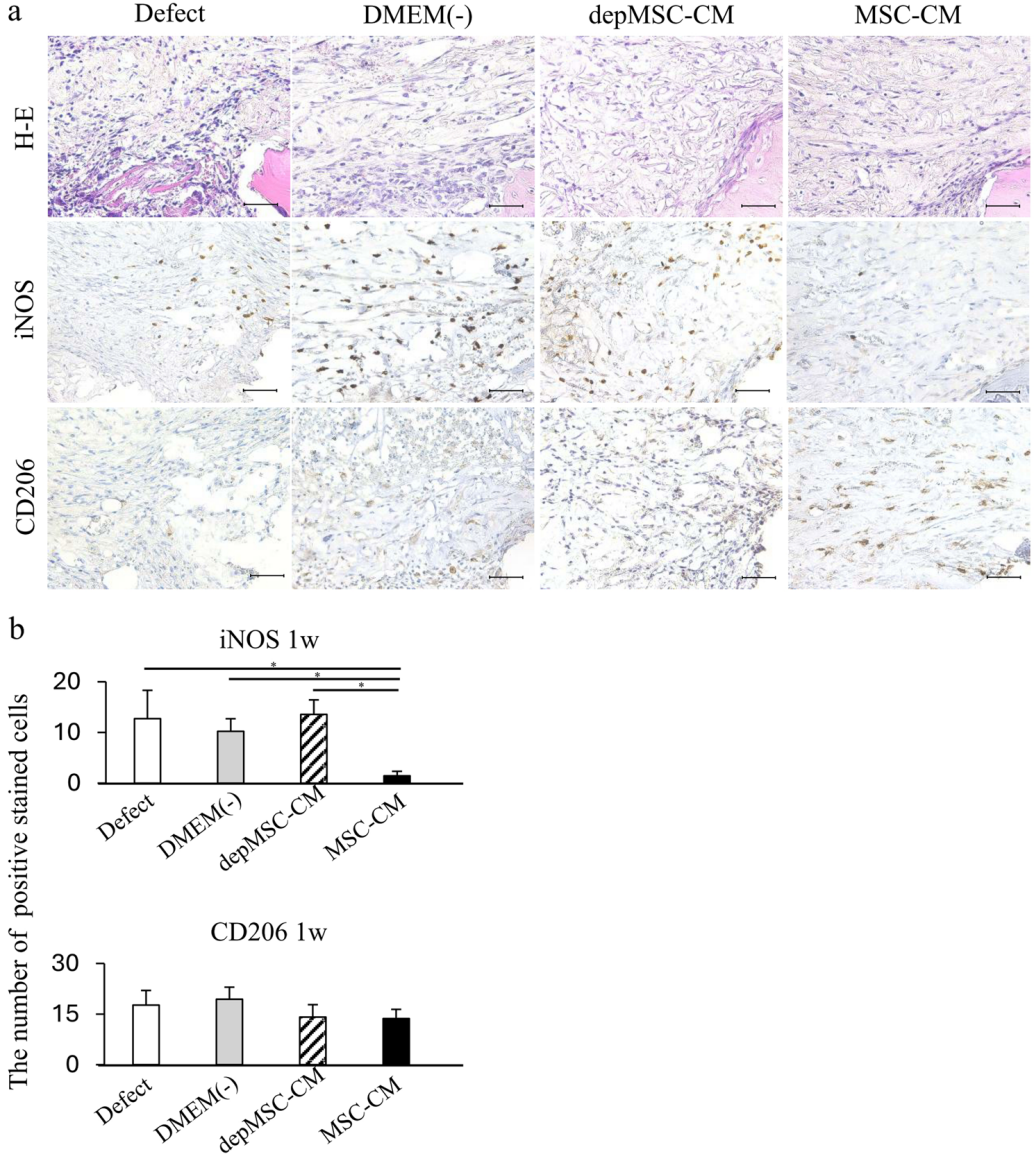
Immunohistochemistry for the newly regenerated bone 1 week after implantation. After 1 week, iNOS-positive cells were few in the MSC-CM group; however, there were no significant differences in the number of CD206 positive cells between the groups (**a**, **b**). Scale bar: 50 μm

**Fig. 8:**
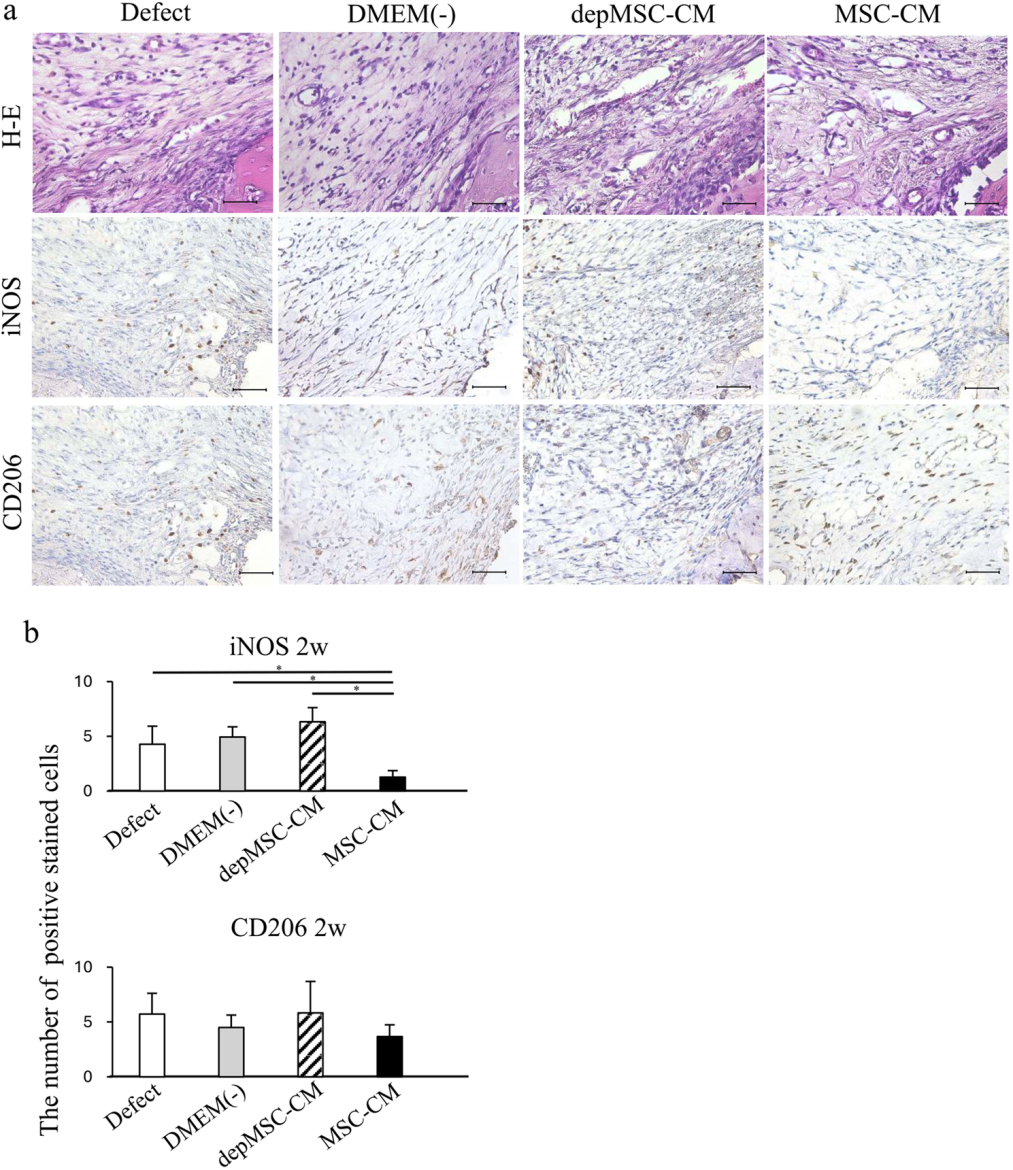
Immunohistochemistry for the newly regenerated bone two weeks after implantation. After 2 weeks, the number of iNOS-positive cells was lower in the MSC-CM group; however, there were no significant differences in the number of CD206 positive cells between the groups (**a**, **b**). Scale bar: 50 μm

Histological analysis also showed that MSC-CMs increased bone regeneration compared with the depMSC-CM, DMEM(-), and defect groups. Seventy-two hours after implantation, migrated inflammatory cells were found around the defect margins in the MSC-CM and depMSC-CM groups; however, bone regeneration was not obvious in any of the groups (Fig. 6a). After 1 and 2 weeks, MSC-CM increased the bone compared to the other groups, and bone formation along with bone defect margins was observed in the MSC-CM group (Figs. 7a and 8a).

### MSC-CM induced macrophage phenotype polarization at an early phase of bone regeneration

**Fig. 9:**
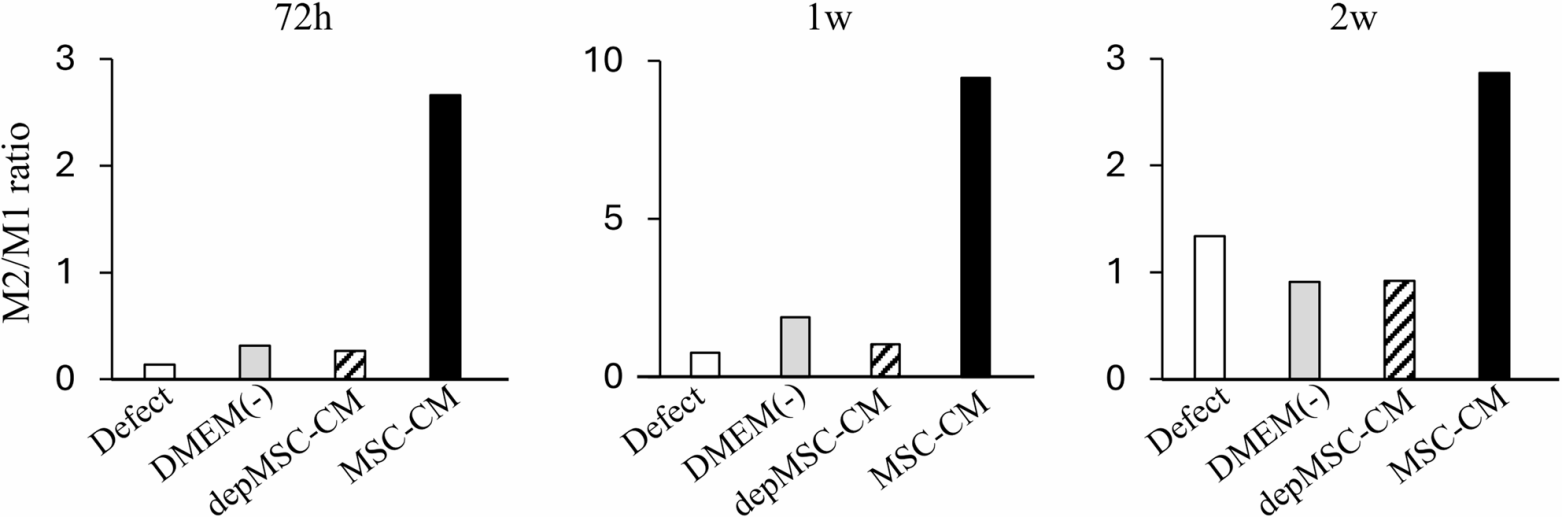
The ratio of CD206-positive (M2) and iNOS-positive (M1) cells after implantation. At Seventy-two hours, one week and two weeks after implantation, MSC-CM improved the M2/M1 ratio around the newly formed bone edge, while it remained low in the other three groups

Cells stained with the pro-inflammatory M1 macrophage marker iNOS and anti-inflammatory macrophage marker CD206 were observed around the margin of bone defect at 72 h, 1 week, and 2 weeks after implantation to investigate macrophage phenotype polarization at an early phase of bone regeneration. After 72 h, the number of iNOS-positive cells in the MSC-CM group (5.6 ± 1.9) was significantly lower than that in the depMSC-CM (17.4 ± 4.1), DMEM(-) (13.4 ± 2.4), and defect (20.0 ± 4.9) groups. In contrast, the number of CD206-positive cells in the MSC-CM group (14.8 ± 2.3) was significantly higher compared to that in the depMSC-CM (4.6 ± 1.6), DMEM(-) (4.2 ± 1.4), and defect (2.7 ± 1.3) groups at 72 h after implantation (Fig. 5a, b). After 1 week, the number of iNOS-positive cells in the MSC-CM group (1.4 ± 0.9) was significantly lower compared to that in the depMSC-CM (13.6 ± 2.9), DMEM(-) (10.2 ± 2.5), and defect (12.8 ± 5.6) groups. However, significant difference in the number of CD206 positive cells between each group was not obvious (Fig. 7a, b). After 2 weeks, the number of iNOS-positive cells was significantly lower in the MSC-CM group (1.3 ± 0.6) compared to that in the depMSC-CM (6.3 ± 1.3), DMEM(-) (4.9 ± 0.9), and defect (4.3 ± 1.6) groups. In contrast, no significant difference in the number of CD206 positive cells was found between each group (Fig. 8a, b). From 72 h to 2 weeks, the M2/M1 ratio was higher in the MSC-CM group than that in the other groups.(Fig. 9).

## Discussion

Since the bone-regenerative effect of MSC-CM was first reported, numerous studies have attempted to elucidate the underlying mechanism of its therapeutic effects. To date, several cytokines in MSC-CM and their effects on bone regeneration have been revealed. Previous studies have reported that VEGF, IGF-1, and TGF-β, which were contained in MSC-CMs, promote bone regeneration through cell proliferation, cell recruitment, and angiogenesis and VEGF as a key factor in tissue regeneration that enhances capillary formation, thus supporting cell migration and blood supply to damaged tissues [7, 16]. Recently, the immune regulatory effects of MSC-CMs through macrophage phenotype polarization and subsequent anti-inflammatory effects have been reported by several studies [11, 12]. We planned to identify the macrophage phenotype-polarization factor in MSC-CM and its effect on bone regeneration in this study.

Macrophages are activated by two major pathways (classical and alternative) and perform a wide range of functions [13]. Classically activated macrophages (also known as pro-inflammatory M1 macrophages) are induced by microbial products, T cell-derived signals, and foreign substances. They actively ingest and produce cytokines that stimulate inflammation, thereby playing an essential role in host defense against chronic inflammatory diseases. Alternatively, M2 macrophages are activated by interleukin (IL)-4 and IL-13 produced by T and mast cells. These macrophages produce TGF-β and other growth factors to terminate inflammation and enter the tissue regenerative phase [11, 17, 18]. Therefore, macrophage phenotype polarization is the principal process for resolving inflammation and initiating tissue regeneration.

MCP-1 is a chemokine that promotes chemotaxis of immune cells and plays a crucial role in inflammation and pathological circumstances [19]. Recently, the ability of MCP-1 to convert the macrophage phenotype towards the M2 phenotype has been reported. Hernan et al. reported that MCP-1 promotes macrophage phenotype polarization towards the M2 type through inhibition of apoptosis and caspase 8 cleavage [20]. In this study, we confirmed that the MSC-CM contained MCP-1 at a concentration of 414.9 ± 138.2 pg/mL. Therefore, we hypothesized that MCP-1 plays a central role in MSC-CM-induced macrophage phenotypic polarization.

MSC-CM-induced immunoregulatory effects and subsequent tissue regeneration are closely associated with macrophage phenotype polarization. Gao et al. reported that MSC-CMs reduced the expression level of tumor necrosis factor (TNF)-α but enhanced the expression levels of IL-10, arginase-1, and CD206 in mouse macrophage RAW264.7 cells. In addition, MSC-CM activated signal transducer and activator of transcription (STAT) 3 but inhibited nuclear factor (NF)-κB pathways in RAW264.7 cells [21]. Our previous report revealed that MSC-CM enhanced macrophage phenotype polarization within 72 h after MSC-CM implantation and promoted bone regeneration in rat calvaria bone defects [12]. In this study, using BMMs in vitro, we revealed that the expression levels of M2 macrophage markers (*CD206* and *Arg-1*) were significantly upregulated, whereas the expression levels of M1 macrophage markers (*iNOS* and *CD80*) were significantly downregulated in the MSC-CM group compared to those in the depMSC-CM group (Fig. 2) Therefore, MCP-1 in MSC-CM is likely to induce macrophage phenotype polarization towards the M2 type. In addition, MSC-CM increased the number of M2 macrophages around the bone edge 72 h after MSC-CM implantation compared to the depMSC-CM group in vivo. Interestingly, the number of M1 macrophage in the MSC-CM group was significantly smaller compared to that in the depMSC-CM group. However, after 1 and 2 weeks, there was no significant difference between the number of M2 macrophages. These results suggest that MSC-CM promote macrophage phenotype polarization towards the M2 type within 72 h after implantation.

The interaction between MSCs and M2 macrophages is a key component in bone regeneration. Gong et al. previously reported that M2 macrophages accelerate MSCs differentiation into osteoblasts by releasing pro-regenerative cytokines (such as TGF-β, VEGF, and IGF-1), whereas M1 macrophages suppress osteoblast cell differentiation by producing pro-inflammatory cytokines (including IL-6, IL-12, and TNF-α) [22]. In our study, MSC-CM enhanced osteogenesis-related gene expression in MSCs. However, the expression levels of *OPN* and *ALP* were significantly downregulated in the depMSC-CM group compared to those in the MSC-CM group in vitro (Fig. 3). These results indicate that MCP-1 in MSC-CM contributes to MSC osteogenesis. In a rat calvaria bone defect model, MSC-CM increased the number of M2 macrophages around the bone edge 72 h after implantation and maintained a high M2/M1 ratio for 2 weeks, resulting in enhanced bone regeneration. In contrast, depMSC-CM did not increase the M2/M1 ratio and subsequent bone regeneration was not obvious (Figs. 4, 5, 6, 7 and 8). Overall, these results indicate that MSC-CM promotes bone regeneration not only via a direct effect on MSCs themselves but also via MCP-1-induced macrophage phenotype polarization towards the M2 type within 72 h after implantation.

This possibility was partially supported by the previous report studying the paracrine effects of murine muscle-derived stem cells (MDSCs) [23]. This study indicated that MDSCs secreted MCP-1 and initiated early inflammation in the bone defect and earlier inflammation resolution, enhanced angiogenesis, and suppressed initial immune response. MCP-1 attracted host macrophage more potently in the bone environment and therefore transition into M2 macrophage stage earlier and promote bone regeneration. Which may explain why M2 number was higher in MSC-CM group only within 72 h after implantation.

MSC-CM contains numerous cytokines, some of which have been addressed in our previous studies to elucidate the underlying mechanism of bone regeneration induced by MSC-CM. We demonstrate the positive effects of MCP-1 on macrophage phenotypic polarization and subsequent bone regeneration. However, the precise mechanism underlying MCP-1-induced macrophage phenotypic polarization and the mechanism by which M2 macrophages promote bone regeneration remain elucidated. Clarifying these mechanisms will contribute to establishing the clinical use of MSC-CM not only for bone regeneration, but also in other inflammatory diseases and bone-resorptive diseases in the oral and maxillofacial regions.

## Conclusions

MCP-1 seemed to be one of the most important contributing factors in MSC-CM-induced macrophage phenotypic polarization at an early stage of bone regeneration and enhanced subsequent bone regeneration.

## Data Availability

The datasets during and/or analyzed during the current study are available from the corresponding author on reasonable request.

## Abbreviations

MSC: Mesenchymal stem cell
MSC-CM: the conditioned medium of human mesenchymal stem cells derived from bone marrow
IGF-1: Insulin like growth factor-1
VEGF: Vascular endothelial growth factor-A
TGF-β1: Transforming growth factor-β1
MCP-1: Monocyte chemoattractant protein-1
DMEM: Dulbecco’s modified Eagle’s medium
ELISA: Enzyme-linked immunosorbent assay
BMM: Bone marrow macrophage
PFA: Paraformaldehyde Phosphate Buffer Solution
FBS: Fetal bovine serum
iNOS: inducible nitric oxide synthase
RT-PCR: Real-Time Quantitative Reverse Transcriptase-Polymerase Chain Reaction
Arg-1: Arginase-1
OPN: Osteopontin
COL I: Type I collagen
ALP: Alkaline phosphate
OCN: Osteocalcin
GAPDH: Glyceraldehyde-3-phosphate dehydrogenase
Micro-CT: Microcomputed Tomography
